# Cyclosporine A for the treatment of refractory nephrotic syndrome with renal dysfunction

**DOI:** 10.3892/etm.2013.1446

**Published:** 2013-12-12

**Authors:** XIANG GAO, YIYI MA, LIJUN SUN, DONGPING CHEN, CHANGLIN MEI, CHENGGANG XU

**Affiliations:** Department of Medicine, Kidney Institute of PLA, Changzheng Hospital, Second Military Medical University, Shanghai 200003, P.R. China

**Keywords:** cyclosporine A, refractory nephrotic syndrome, renal dysfunction, therapy

## Abstract

Cyclosporine A (CsA) is an immunosuppressant agent and is utilized as a second-line drug therapy for refractory nephrotic syndrome (RNS). In general, the use of CsA is strictly controlled in patients with an estimated glomerular filtration rate (eGFR) <30–40 ml/min/1.73 m^2^, and little is known about the safety and efficacy of CsA treatment in patients with RNS complicated by renal dysfunction. In the present study, the clinical data of 10 patients with RNS and renal dysfunction, who received CsA treatment between 2000 and 2009 in the Kidney Institute of PLA, were reviewed retrospectively. Pathologically, these patients included six cases with minimal change, two cases of diffuse mesangial proliferation and two cases of focal segmental glomerulosclerosis. Six months subsequent to the initiation of the CsA treatment, six patients achieved complete remission, two patients achieved remarkable remission and two patients achieved partial remission. Renal function was improved in all patients as represented by the improvement in the eGFR (28.6±3.8 ml/min/1.73 m^2^ prior to treatment versus 99.3±21.9 ml/min/1.73 m^2^ 6 months subsequent to treatment). Few adverse CsA-related events were observed. These results suggest that renal dysfunction is not an absolute contraindication for CsA treatment in patients with RNS. The use of CsA is safe and efficacious and may, in certain cases, improve renal function in patients with RNS and renal impairment.

## Introduction

Refractory nephrotic syndrome (RNS) is characterized as a steroid-dependent, steroid-resistant or frequently relapsing nephrotic syndrome, and the majority of pathological types of idiopathic nephrotic syndrome can present as RNS (1,2). Cyclosporine A (CsA) is an immunosuppressant agent that is widely used in clinical practice, predominantly for the prevention of rejection in various types of organ transplantation (3,4). In addition, CsA is considered to be the second-line drug therapy for RNS (5). The use of CsA is hampered by several problems, and baseline renal function is considered to be an important prerequisite in RNS (6). In general, the use of CsA is strictly controlled in patients with an estimated glomerular filtration rate (eGFR) <30–40 ml/min/1.73 m^2^(6,7). However, an improvement in renal function may be observed in patients who have undergone an organ transplant following a CsA dose reduction, and there is little information on patients with RNS and impaired renal function (8,9). In the present study, the use of CsA was reviewed in 10 patients with RNS and renal dysfunction in the Kidney Institute of PLA (Shanghai, China) to evaluate the safety and efficacy of CsA in specific cases.

## Patients and methods

### Patient characteristics

Data were collected retrospectively from the inpatient records of the Kidney Institute of PLA between 2000 and 2009, and a total of 10 patients with RNS and renal dysfunction were included. The baseline characteristics of the patients are listed in Table I. The clinical presentations of the patients included nephrotic syndrome, large quantities of protein in the urine and severe edema.

### Treatment protocol

All the cases were diagnosed as RNS, including seven cases of steroid-resistant RNS and three cases of steroid-dependent RNS. The treatment protocol comprised CsA with low-dose steroids. The mean initial dose of CsA (Neoral^®^, 25 mg; Novartis Pharma Schweiz AG, Basel, Switzerland) was 3.08±0.22 mg/kg/day, in combination with low-dose steroid therapy (prednisone, 0.5 mg/kg/day; Shanghai Xinyi Pharmaceutical Co., Ltd,, Shanghai, China; or methylprednisolone, 0.4 mg/kg/day; Pfizer Ltd., Kent, UK). The dose of CsA was adjusted during the follow-up period by controlling the drug concentration within 100–200 ng/ml. Anticoagulation, blood pressure control and gastric mucosal-protective and osteoporosis-preventative therapies were also prescribed.

### Definitions, follow-up and safety assessment

The efficacy of CsA was evaluated by the remission status as: (i) Complete remission, which was defined as the absence of symptoms, 24-h urine protein <0.3 g and serum albumin ≥35 g/l; (ii) remarkable remission, which was defined as the absence of symptoms, 24-h urine protein <1.5 g and serum albumin ≥30 g/l; and (iii) partial remission, which was defined as the absence of the majority of the symptoms, a 50% decease in 24-h urine protein (although remaining at >1.5 g) and serum albumin <30 g/l. Inefficacy was defined as the symptoms remaining uncontrolled, a <50% decrease in 24-h urine protein and serum albumin <30 g/l (10).

The patient status was evaluated at 0, 1, 3 and 6 months following the initiation of the CsA therapy. The observational outcome data were collected and analyzed, including the blood CsA concentration, urine output, body weight, 24-h urine protein excretion, albumin and eGFR, as determined by the Modification of Diet in Renal Disease (MDRD) equation prior to and following the intervention. The safety of CsA use was evaluated by changes in blood pressure (BP), blood glucose (BG), serum creatinine (Scr) and alanine aminotransferase level (ALT) prior to and following the intervention.

### Ethical approval

The study protocol was approved by the Ethics Committee of the Second Military Medical University (Shanghai, China). Written informed consent was obtained from all the patients involved. The study was conducted in accordance with the Declaration of Helsinki and national laws.

### Statistic analysis

The results are expressed as the mean ± standard deviation. The data prior to and following the treatment were compared using a paired-samples t-test. The statistical analysis was performed using StatView 4.0 software (Abacus Concepts, Inc., Berkeley, CA, USA). P<0.05 was considered to indicate a statistically significant difference.

## Results

### Efficacy

Clinical efficacy was observed in all 10 patients one month after the start of the CsA treatment, with the results demonstrating an increase in urine output and serum albumin and a decrease in body weight. Three months after the initiation of the treatment, four patients achieved complete remission, four patients achieved remarkable remission and two patients achieved partial remission. Six months after the start of the treatment, six patients achieved complete remission, two patients achieved remarkable remission and two patients achieved partial remission. The edema disappeared in all of the patients. The clinical parameters prior to and following the interventions are shown in Table II.

### Safety

No drug-related adverse events occurred during the six-month treatment period. The BP, BG and liver/kidney function prior to and following treatment are shown in Table III. BP and BG were not found to be significantly different prior to and following the initiation of treatment. However, renal function was significantly improved following treatment. The ALT levels were observed to be significantly elevated in specific cases, although they remained within the normal range, indicating that there was no significant impairment to the liver function of the patients.

## Discussion

CsA is an immunosuppressive agent that is able to selectively target T lymph cells, inhibit the production of interleukin-2 (IL-2) and the expression of the IL-2 receptor, reduce the production and accumulation of cytokines, alleviate the inflammatory reaction, improve the size and charge selectivity of the glomerular basement membrane, reduce filtration and promote the reconstruction of foot processes to reduce the concentration of urine protein and suppress the proliferation of mesangial cells (11,12). CsA was first used to treat idiopathic glomerular disease associated with nephrotic syndrome in adults in 1985. Previous studies have demonstrated the efficacy of CsA in the treatment of RNS (5,7,13,14). However, the toxicity of CsA is significant. The underlying mechanisms include acute renal toxicity by the activation of the renin-angiotensin system and the fibrogenic effect contributing to chronic renal interstitial fibrosis (15,16). Renal function and the drug dosage are important factors affecting the toxicity of CsA (17).

All the 10 cases in our study were RNS patients with impaired renal function, of whom seven patients had an Scr level >200 μmol/l. The mean eGFR of the 10 cases, as assessed using the MDRD equation, was 28.6±3.8 ml/min/1.73 m^2^. From the aspect of general immune suppressive therapy, it is necessary for CsA to be strictly controlled or even avoided under this condition. However, considering the renal pathological features of the 10 cases (open capillary loops, mild tubulointerstitial changes, marked swelling of the tubule epithelial cells and protein casts, and the absence of severe focal or patchy necrosis), we hypothesized that the main mechanism of renal dysfunction was the lack of effective circulating volume and the edema/obstruction of the renal tubulointerstitum, leading to a decrease in renal Scr excretion. In view of the dose-dependent renal toxicity of CsA and the efficacy of low-dose steroids in improving the therapeutic effect of CsA, we proposed that the combination of low-dose CsA and steroids was likely to be efficacious and safe for the protection of renal function, based on the improved effective circulating volume and dialysis where necessary (18). In total, 7 of the 10 patients received bedside slow continuous ultrafiltration (SCUF), in which albumin was used during the SCUF to reduce edema and achieve an effective circulating volume. Two patients received intermittent albumin/plasma/dextran-amino acid plus furosemide combination therapy. In addition, one patient received continuous renal replacement therapy (continuous veno venous hemodiafiltration) for five days and no progressive Scr increase was observed following the discontinuation of the therapy. The anticoagulation therapy lasted ≥1 month, during which intravenous heparin or subcutaneous low molecular weight heparin was used alternately. The dose of heparin was reduced after the urine protein was reduced. The patients were also administered with therapies to regulate lipid metabolism and protect the gastric mucosa. BP was controlled using antihypertensive drugs. The initial dose of CsA was controlled within 3.5 mg/kg/day, based on the increased body weight caused by severe edema.

One week subsequent to the initiation of treatment, the Scr levels increased mildly by <30% in all 10 patients, and there were no statistical differences compared with the baseline values. The Scr levels then began decreasing gradually and were reduced to the normal range 6 months after the initiation of treatment. The urine output increased, with no acute exacerbation of oliguria or anuria. One month after the initiation of treatment, the 24-h urine output was >1,000 ml in all patients. Based on the gradual decrease in the 24-h urine protein excretion and an increase in serum albumin levels, the 6-month treatment outcome showed 100% efficacy and 60% complete remission. No significant drug-related adverse events were observed. Although the ALT levels increased in a few cases, the levels remained within the normal range and no symptoms of liver dysfunction were observed.

In conclusion, CsA appears to be safe and efficacious in the treatment of RNS. Impaired renal function may not be the absolute contraindication for CsA use and renal pathological changes and causes of renal dysfunction may be more important determinants. In the absence of severe glomerular and tubulointerstitial proliferation or necrosis, decreased renal perfusion may be the main cause of renal function impairment. Therefore, on the basis of an improvement in renal perfusion, CsA can preserve or even improve renal function in RNS patients with renal impairment. Further studies, involving larger numbers of patients and longer follow-up periods, are required to prove the long-term safety and efficacy of CsA therapy for patients with RNS and renal dysfunction.

## Figures and Tables

**Table I:** Baseline characteristics of the patients.

Parameter	Value
Cases, n	10
Gender, n	
Female	6
Male	4
Pathological change, n	
Minimal disease change	6
Diffuse mesangial proliferation	2
Focal segmental glomerulosclerosis	2^a^
Mean age, years	36.3±11.7
Mean course of disease, months	6.2

a 1/15 and 2/22 focal segmental changes in the two cases of focal segmental glomerulosclerosis.

**Table II:** Comparison of clinical parameters prior to and following cyclosporine A treatment.

Time-point	Drug concentration, ng/ml	Body weight, kg	24-h urine output, ml/day	24-h urine protein, mg/day	Albumin, g/l	eGFR, ml/min/1.73 m^2^
Prior to therapy	-	69.7±6.7	390.0±60.0	5652.6±2202.2	16.9±1.8	28.6±3.8
Following therapy						
1 month	154.4±19.1	63.1±6.4^a^	1547.0±136.7^a^	2128.2±2006.4^a^	24.8±2.5^a^	67.4±12.9^a^
3 months	151.0±17.2	58.8±6.7^a^	1573.5±305.4^a^	991.6±1363.2^a^	33.3±5.2^a^	105.5±30.3^a^
6 months	139.0±25.3	56.6±5.9^a^	1614.0±237.4^a^	555.5±737.5^a^	38.0±4.5^a^	99.3±21.9^a^

Data prior to and following the treatment were compared using a paired-samples t-test.

a P<0.01, vs. value prior to therapy.

eGFR, estimated glomerular filtration rate.

**Table III:** Laboratory parameters concerning adverse events prior to and following cyclosporine A treatment.

Time-point	BP, SBP/DBP mmHg	Scr, μmol/l	ALT^c^, U/l	Blood glucose, mmol/l
Prior to therapy	129.5±10.6/86.0±5.2	208.4±31.2	17.0±8.0	4.9±0.4
Following therapy				
1 week	132.5±12.7/87.5±7.9	217.8±29.9	22.8±6.5^a^	-
2 weeks	133.0±11.4/82.5±5.9	164.7±17.1^b^	-	-
1 month	131.5±11.1/81.0±4.6	99.5±8.8^b^	23.8±7.0^a^	4.7±0.5
3 months	129.0±8.8/76.5±6.7	69.4±12.7^b^	23.3±5.5^a^	4.8±0.4
6 months	127.0±5.4/75.0±5.3	71.4±6.6^b^	27.8±3.9^a^	5.0±0.4

Data prior to and following cyclosporine A treatment were compared using a paired-samples t-test.

a P<0.05 and

b P<0.01, vs. the value prior to therapy.

c ALT levels were significantly increased in specific cases but remained within the normal range.

SBP, systolic blood pressure; DBP, diastolic blood pressure; Scr, serum creatinine; ALT, alanine aminotransferase.
